# Pancreatic Myeloid Sarcoma Causing Obstructive Jaundice: A Case Report and Literature Review

**DOI:** 10.1155/2024/5513857

**Published:** 2024-03-11

**Authors:** Laura E. Lavette, Angela G. Niehaus, Clancy J. Clark, Jason D. Conway, Girish Mishra, Darius A. Jahann

**Affiliations:** ^1^Department of Internal Medicine, University of Virginia Medical Center, 1215 Lee St., Charlottesville, Virginia 22903, USA; ^2^Department of Pathology, Wake Forest Baptist Health, Medical Center Blvd., Winston-Salem, North Carolina 27157, USA; ^3^Department of Surgery, Wake Forest Baptist Health, Medical Center Blvd., Winston-Salem, North Carolina 27157, USA; ^4^Department of Medicine, Section on Gastroenterology and Hepatology, Wake Forest Baptist Health, Medical Center Blvd., Winston-Salem, North Carolina 27157, USA

## Abstract

Myeloid sarcoma (MS) is an extramedullary manifestation of acute myeloid leukemia (AML) and commonly occurs in sites such as the lymph nodes, skin, soft tissues, and bone. It more rarely manifests in the pancreas, with less than 20 cases reported in the literature since 1987. Despite its rarity, MS should be considered in the differential diagnosis of a soft tissue mass causing obstructive jaundice, especially if the patient has a known hematologic disease. Isolated cases of pancreatic MS have been known to progress to AML; therefore, it is crucial to differentiate MS from more common diagnoses, such as pancreatic cancer or pancreatitis. This is a case of a 70-year-old male with symptomatic obstructive jaundice secondary to pancreatic MS, ultimately requiring endoscopic ultrasound (EUS) and endoscopic retrograde cholangiopancreatography (ERCP) for diagnosis and management. Also included is a comprehensive review of previous case reports with similar clinical presentations, management, and treatment of pancreatic MS.

## 1. Introduction

First described in 1811, MS is an extramedullary solid tumor arising from myeloid precursor cells. MS may develop in isolation or in association with AML, chronic myeloid leukemia (CML), myeloproliferative, or myelodysplastic disorders [1]. MS has been recognized in approximately 5% of AML patients [2]. Isolated MS, defined by the absence of leukemic blasts in the peripheral blood and bone marrow, is less commonly seen and estimated to affect only 2 out of every 1,000,000 adults with an age-adjusted incidence rate of 0.9% [3]. Importantly, previous studies indicate high rates of misdiagnosis in isolated MS, likely due to its wide-ranging clinical presentation [4]. Delayed diagnosis and treatment may worsen a patient's prognosis and allow for the development of AML [5].

MS is commonly observed in the lymph nodes (15–25%), skin (13–22%), and bone/spine (9–25%) [6, 7]. MS infiltration of the pancreas is particularly rare, with less than 20 cases reported since 1987. The average age of pancreatic MS is 40 years old, with over half of the reported cases being women [5, 8–22]. Clinical manifestations are nonspecific and often vary depending on tumor location and size. Pancreatic MS most commonly presents with abdominal pain and often mimics more common diagnoses, such as pancreatitis or pancreatic cancer. Over half of the reported cases have also been associated with obstructive jaundice; however, only five patients underwent diagnostic EUS with just two requiring ERCP for management of biliary obstruction. Here, we present a rare case of obstructive jaundice secondary to MS of the pancreas. We have summarized cases with similar endoscopic management through a related literature review.

## 2. Case Presentation

**Figure 1:**
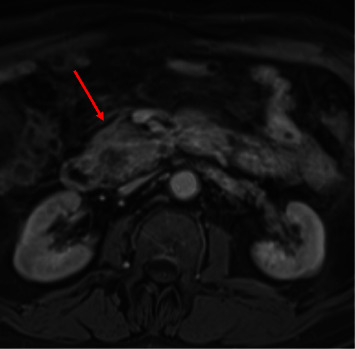
MRI of the abdomen showing ill-defined mass in the inferior pancreatic head/uncinate process with small hypervascular component and large hypovascular component measuring 1.9 × 2.0 cm. There is adjacent side branch ectasia but no dilatation of the main pancreatic duct.

A 70-year-old man with recently diagnosed leukemia cutis of the eyelid presented with one week of epigastric abdominal pain and jaundice. His physical exam was significant for a slightly distended, nontender abdomen with scleral icterus and jaundice. Biochemical data obtained at time of admission included (reference range in parentheses) total bilirubin, 12.1 mg/dL (0.1–1.2 mg/dL); direct bilirubin, 7.8 mg/dL (0.1–0.2 mg/dL); serum aspartate aminotransferase, 261 U/L (5–40 U/L); serum alanine aminotransferase, 435 U/L (5–50 U/L); alkaline phosphatase, 670 U/L (25–125 U/L); white blood cell, 7,600/*μ*L (4,400–11,000/*μ*L); carcinoembryonic antigen, 0.8 ng/mL (0–5 ng/mL); and carbohydrate antigen 19–9, 461.69 U/mL (0–35 U/mL). Magnetic resonance imaging (MRI) illustrated an ill-defined pancreatic mass in the inferior pancreatic head/uncinate process with large hypovascular and small hypervascular components and upstream biliary ductal dilation (Figure 1).

**Figure 2:**
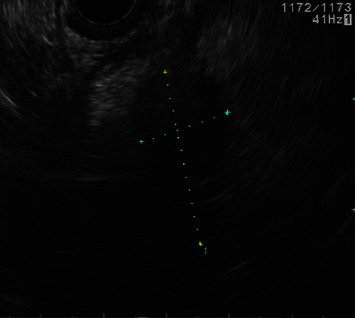
EUS demonstrating a hypoechoic area near the distal CBD/pancreatic head measuring approximately 20 mm.

**Figure 3:**
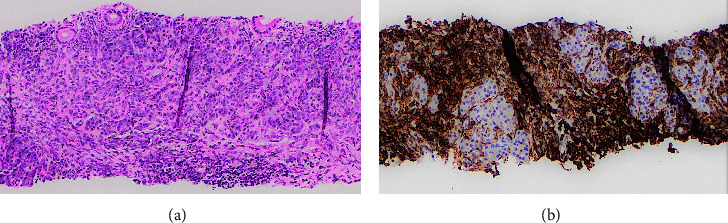
(a) Core needle biopsy with atypical hematopoietic cells. (b) Positive stain for CD45, which is commonly expressed in myeloid sarcomas.

For purposes of tissue acquisition, the patient underwent EUS with fine needle biopsy which demonstrated a 20 mm hypoechoic pancreatic mass near the distal common bile duct (CBD) and pancreatic head (Figure 2). ERCP delineated a high-grade distal biliary stricture with upstream dilation for which a complete biliary sphincterotomy was performed and a plastic stent was placed. The core sample illustrated atypical hematopoietic cells dissecting through the pancreatic parenchyma, immunoreactive for MPO and CD45, confirming a diagnosis of myeloid sarcoma (Figure 3). Genetic sequencing of the tumor ultimately revealed presence of *IDH1*, isocitrate dehydrogenase, and gene mutation.

At the time of diagnosis, there was no evidence of bone marrow involvement or systemic disease. Four days following ERCP, the patient began induction chemotherapy with a standard induction of “7 + 3” which involved cytarabine (200 mg/m^2^) continuous infusion for 7 days with daunorubicin (12 mg/m^2^) for 3 days. A positron emission tomography (PET) scan two weeks later showed persistent hypermetabolic soft tissue involving the pancreatic head, suggesting disease progression and requiring high-dose cytarabine plus mitoxantrone. Following completion of reinduction therapy, the patient's PET scan and bone marrow biopsy showed no residual or recurrent disease. The patient then underwent successful allogeneic stem cell transplant.

**Figure 4:**
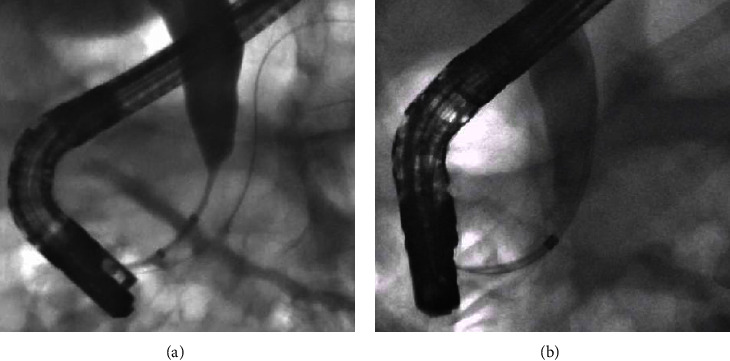
(a) Initial ERCP showing a high-grade distal biliary stricture. (b) Following chemotherapy, repeat ERCP illustrates resolution of the stricture.

Three months after the first ERCP, the patient returned for repeat ERCP and stent revision. The previous distal biliary stricture from MS had completely resolved, and the patient did not require further biliary prosthesis (Figure 4).

## 3. Discussion

Pancreatic MS is an extramedullary solid tumor that most commonly presents with abdominal pain and obstructive jaundice. This nonspecific presentation can easily mimic a pancreatic cancer, especially in the absence of a concurrent hematologic disorder that may indicate systemic disease. Ductal adenocarcinoma accounts for 85% of pancreatic neoplasms and is often the leading concern in older patients with obstructive jaundice [23, 24]. Other common differentials include cholelithiasis, strictures, or infection/inflammation [25]. The initial approach to obstructive jaundice includes liver function tests and cross-sectional imaging, such as CT or MRI [26]. EUS may be required for staging and tissue acquisition when looking for a definitive diagnosis. ERCP can also be utilized in (1) a diagnostic fashion via brushings for cytology of malignant strictures or single operator cholangioscopy with biopsies and (2) a therapeutic role for relief of biliary obstruction. Our case describes a rare cause of obstructive jaundice, MS of the pancreas, and underscores the utility of EUS and ERCP in diagnosis and management.

Although ductal adenocarcinoma is a common culprit of obstructive jaundice, less common diagnoses should also be considered, including MS. Pancreatic MS is rare, with only 18 cases currently reported in the literature [5, 8–22]. We conducted a literature search using the PubMed database and identified publications relevant to pancreatic MS. Our search included the key words “pancreas,” “myeloid sarcoma,” “chloroma,” and “granulocytic sarcoma.” After individually reviewing cases and cross-referencing recent literature reviews, we compiled all reports of pancreatic MS dating back to 1987. Among these 18 case reports, 11 were female (61%) and 7 were male (39%). The mean age of diagnosis was 40 years (ranging from 15 to 75 years), with 10 cases describing isolated MS (56%). Fourteen (78%) patients' neoplasms were located in the pancreatic head, while the remainder (22%) were found in the pancreatic tail and body. The most common presenting symptoms included abdominal pain (89%) and obstructive jaundice (61%). Despite over half of the patients developing obstructive jaundice, only five underwent EUS and two required ERCP (Table 1). Compared to previously published case reports, we believe that our work is the third and oldest patient with pancreatic MS to require ERCP reported in the literature. Our case also provides additional context regarding the utility of EUS in timely, accurate diagnosis and the value of early chemotherapy induction. Regarding the findings on EUS, there is no consensus in the literature about the endosonographic appearance of a myeloid sarcoma compared to a pancreatic adenocarcinoma. In our case, it was noted that the lesion did have relatively well-demarcated borders which is atypical for the majority of pancreatic cancer. In contrast, in any pancreatic mass causing a biliary obstruction, whether myeloid sarcoma or adenocarcinoma, ERCP findings on cholangiogram are similar and illustrated by a high-grade stricture at the site of ductal obstruction.

The first case of pancreatic MS requiring ERCP was published in 1999, in which a 31-year-old male was initially misdiagnosed with pancreatitis via EUS-guided fine needle aspiration (FNA) [14]. Due to persistent symptoms, the patient underwent laparotomy with open biopsy. He was confirmed to have pancreatic MS, for which he was promptly started on chemotherapy with nearly complete resolution of the pancreatic mass. A more recent case from 2018 described a 36-year-old male who was similarly misdiagnosed with pancreatitis [17]. The patient eventually underwent a more extensive work-up, including EUS, ERCP, PET-CT, and ultrasound-guided FNA, which provided a diagnosis of MS. Chemotherapy was initiated over a year after onset of symptoms, and the patient ultimately developed AML with unsuccessful treatment of his extramedullary disease.

In the two aforementioned cases, pancreatic MS was initially mistaken for a more common diagnosis and appropriate treatment was delayed. Although there is currently no standard treatment for pancreatic MS due to the rarity of the disease, 16 of the 18 known cases included chemotherapy (±resection or radiotherapy) as part of their treatment strategies. Of those 16, 11 reported more information on the regimen of choice, with all of them being cytarabine-based therapies of varying length and dosage. This is in accordance with the National Comprehensive Cancer Network (NCCN) guidelines recommending systemic therapy for extramedullary disease such as MS. Half of the patients who received chemotherapy had known resolution of their pancreatic masses. The two patients who did not receive chemotherapy passed away, one from the cancer and the other from septic shock. Based on the existing literature and reported patient outcomes, management of MS necessitates a systemic approach as opposed to localized treatment via resection or radiotherapy [27]. Moreover, without chemotherapy, there is a higher likelihood that isolated MS will develop into AML, as seen in the second case reported above [5, 17].

The characteristics of the cases included in our literature review vary widely; however, a key commonality is the importance of early recognition of pancreatic MS and rapid induction of therapy. Our unique case offers two additional learning points: (1) the differential diagnosis for a pancreatic mass ought to include pancreatic MS, especially when there are signs of a coexisting hematologic disorder, and (2) biliary obstruction, when identified early in this disease, should prompt timely EUS and ERCP for diagnosis and relief of the obstruction followed by rapid institution of chemotherapy. Our case not only supports the key findings from our literature review but provides more insight into the advantages of advanced endoscopy in diagnosis, management, and treatment of uncommon causes of obstructive jaundice.

## Figures and Tables

**Table 1:** Clinical characteristics, treatment, and outcomes of previously reported pancreatic MS cases (1–18) and our case (19).

#	Date	Age	Gender	Chief complaint(s)	Coexisting malignancy?	Location	Obstructive jaundice?	EUS?	MRCP/ERCP?	Stent?	Biopsy method	Treatment	Outcome	Citation #
1	1987	36	F	Nausea, jaundice, and abd pain	N	Head	Y	N	N	N	Open	Gastrojejunostomy, cholecystojejunostomy, radiotherapy, and chemotherapy	Resolution	21
2	1996	32	M	Jaundice and abd pain	N	Head	Y	N	N	N	Open	Whipple and chemotherapy	Resolution	5
3	1997	37	F	Jaundice and abd pain	AML	Head	Y	N	N	N	Autopsy	Chemotherapy	Death	8
4	1999	31	M	Abd pain	N	Head	Y	N	ERCP	Y	1. FNA, 2. Open	Chemotherapy	Resolution	14
5	1999	61	F	Abd pain	AML	Head	N	N	N	N	Unknown	Chemotherapy	Death	14
6	2003	42	F	Abd pain and fatigue	AML	Body/tail	N	N	N	N	CT-guided	Chemotherapy	Resolution	13
7	2003	64	M	Diarrhea, abd pain, and jaundice	AML	Head	Y	N	N	N	CT-guided	Percutaneous biliary drains and chemotherapy	Resolution	15
8	2008	75	F	Abd pain, weight loss, epistaxis, and jaundice	AML	Head	Y	N	N	N	Gastric mucosa	Chemotherapy	Resolution	11
9	2010	40	M	Jaundice and weight loss	N	Head	Y	N	MRCP	N	Open	Whipple and chemotherapy	Resolution	9
10	2010	15	F	Jaundice	AML	Head	Y	N	N	N	Autopsy	None	Death	10
11	2011	48	F	Abd pain and fever	N	Body/tail	N	N	N	N	Open	Splenectomy and distal pancreatectomy	Death	18
12	2012	45	F	Abd pain and jaundice	N	Head	Y	Y	N	N	Open	Whipple and chemotherapy	Death	16
13	2012	19	F	Abd pain	N	Head	N	Y	N	N	Open	Resection and chemotherapy	Death	16
14	2016	19	M	Abd pain	AML	Head	N	Y	N	N	EUS-guided FNA	Chemotherapy	Unknown	12
15	2018	34	M	Abd pain	N	Body/tail	N	N	N	N	Laparoscopic	Chemotherapy	Resolution	19
16	2018	36	M	Abd pain	N	Body/tail	Post-EUS	Y	ERCP	Y	1. EUS-guided FNA, 2. U/S-guided FNA	Chemotherapy	Unknown	17
17	2020	57	F	Weakness, abd pain, and jaundice	N	Head	Y	N	N	N	Open	Whipple and chemotherapy	Unknown	22
18	2021	32	F	Abd pain	AML	Head	Y	Y	N	N	1. EUS-guided FNA, 2. U/S-guided FNA	Chemotherapy	Unknown	20
19	2021	70	M	Abd pain and jaundice	AML	Head	Y	Y	ERCP	Y	EUS-guided FNA	Chemotherapy	Resolution	Our case

## Data Availability

Readers can access supporting data by referring to Table 1 and listed references.
